# Associations of Frequency of Laughter With Risk of All-Cause Mortality and Cardiovascular Disease Incidence in a General Population: Findings From the Yamagata Study

**DOI:** 10.2188/jea.JE20180249

**Published:** 2020-04-05

**Authors:** Kaori Sakurada, Tsuneo Konta, Masafumi Watanabe, Kenichi Ishizawa, Yoshiyuki Ueno, Hidetoshi Yamashita, Takamasa Kayama

**Affiliations:** 1 Department of Fundamental Nursing, Yamagata University Faculty of Medicine School of Nursing, Yamagata, Japan; 2 Department of Public Health and Hygiene, Yamagata University Graduate School of Medical Science, Yamagata, Japan; 3 Global Center of Excellence Program Study Group, Yamagata University School of Medicine, Yamagata, Japan

**Keywords:** laughter, mortality, cardiovascular disease, Yamagata study

## Abstract

**Background:**

Positive and negative psychological factors are associated with mortality and cardiovascular disease. This study prospectively investigated associations of daily frequency of laughter with mortality and cardiovascular disease in a community-based population.

**Methods:**

This study included 17,152 subjects ≥40 years old who participated in an annual health check in Yamagata Prefecture. Self-reported daily frequency of laughter was grouped into three categories (≥1/week; ≥1/month but <1/week; <1/month). Associations of daily frequency of laughter with increase in all-cause mortality and cardiovascular disease incidence were determined using Cox proportional hazards modeling.

**Results:**

During follow-up (median, 5.4 years), 257 subjects died and 138 subjects experienced cardiovascular events. Kaplan-Meier analysis revealed that all-cause mortality and cardiovascular disease incidence were significantly higher among subjects with a low frequency of laughter (log-rank *P* < 0.01). Cox proportional hazard model analysis adjusted for age, gender, hypertension, smoking, and alcohol drinking status showed that risk of all-cause mortality was significantly higher in subjects who laughed <1/month than in subjects who laughed ≥1/week (hazard ratio [HR] 1.95; 95% confidence interval [CI], 1.16–3.09). Similarly, risk of cardiovascular events was higher in subjects who laughed ≥1/month but <1/week than in subjects who laughed ≥1/week (HR 1.62; 95% CI, 1.07–2.40).

**Conclusion:**

Daily frequency of laughter represents an independent risk factor for all-cause mortality and cardiovascular disease in a Japanese general population.

## INTRODUCTION

Previous studies have revealed that positive psychological factors promote longevity and reduce the risk of cardiovascular disease, whereas physical disability and negative psychological factors, such as depression, anxiety, and psychological distress, are associated with increased risk of coronary heart disease and cerebrovascular disease.^1^^,^^2^ In Japan, similar findings have been observed, such as associations between perceived level of life enjoyment^3^ and positive psychological factors, such as “ikigai”,^4^ with risk of cardiovascular disease incidence and mortality. In a series of centenarian studies, lower levels of neuroticism and higher levels of extraversion, competence and trust,^5^ openness, conscientiousness and extraversion^6^ were often observed among centenarians. In addition, a positive attitude towards life (PATL), such as an easygoing nature, optimism, laughter, and extraversion/outgoing behavior have shown high inter-correlations among Ashkenazi Jewish centenarians, with PATL and emotional expression showing adequate levels of internal consistency.^7^ The idea that humor and laughter have positive health benefits has become increasingly popular in recent years, among both healthcare providers and the general public. Hayashi et al analyzed cross-sectional data in 20,394 individuals aged 65 years or older who participated in the Japan Gerontological Evaluation Study in 2013. They showed that a higher daily frequency of laughter was associated with lower prevalence of cardiovascular diseases among older Japanese adults.^8^ Due to its cross-sectional nature, however, their study did not clearly show any preventive effect of laughter on cardiovascular disease. No longitudinal prospective study of the daily frequency of laughter and all-cause mortality and cardiovascular disease incidence has yet appeared.

Here, we prospectively investigated the association between daily frequency of laughter and all-cause mortality and cardiovascular disease incidence in a community-based population.

## MATERIALS AND METHODS

The Yamagata Study was a community-based prospective cohort study that was a component of a molecular epidemiological study which utilized the regional characteristics of a 21st century Center of Excellence (COE) program and the Global COE program in Japan.^9^^–^^13^ This study was approved by the ethics committee of Yamagata University School of Medicine (23 Oct 2018, 2018-276). All participants provided written informed consent prior to enrolment. The procedures were performed in accordance with the Declaration of Helsinki. The studied subjects were participants in a community-based annual health check, in which residents of seven cities (Yamagata, Sakata, Kaminoyama, Sagae, Higashine, Yonezawa, and Tendo) in Yamagata Prefecture, Japan, aged 40 years or older were invited to participate. This study had no exclusion criteria. From 2009 through to 2015, a total of 20,969 subjects (8,558 males, 12,411 females) were enrolled. The number of potential subjects was 28,528. During the follow-up period, 66 subjects were lost to follow-up because they moved to other areas.

Participants were followed for up to 8 years (median, 5.4 years) and examined for associations between daily frequency of laughter and all-cause mortality and cardiovascular disease incidence. We excluded 3,817 subjects from analysis because of incomplete data at baseline. Data from a total of 17,152 subjects (7,003 males, 10,149 females) were ultimately entered into the final statistical analysis.

### Measurements

At baseline, survey subjects were mailed a self-reported questionnaire to document their medical history, current medications and clinical symptoms, blood pressure, frequency of laughter, alcohol drinking status, smoking status, physical activity, education level, marital status, level of perceived mental stress, and social participation.

Daily frequency of laughter was assessed via a single-item question: “How often do you laugh out loud?”.^14^ We defined ‘laugh out loud’ as laughter. We provided four possible answers, namely, almost every day, 1–5 times/week, 1–3 times/month, and <1 time/month, and did not allow responses as free descriptions. Self-reported daily frequency of laughter was grouped into three categories (≥1/week; ≥1/month but <1/week; or <1/month). Because the Kaplan-Meier curves for laughter almost every day and laughter 1–5 times/week were similar, we analyzed the data of these groups as one group. We selected “laughter ≥1/week” as the reference category. Alcohol drinking status was classified into three categories: current drinker, past drinker, or nondrinker. Smoking status was classified into the three categories of current smoker, past smoker, or nonsmoker. We inquired about the frequency of participation in different civic associations and social groups. Frequency of social participation was classified into three categories: ≥1/week; ≥1/month but <1/week; or <1/month. Perceived mental stress was evaluated using a single question according to the Japan Collaborative Cohort Study: ‘Did you feel mental stress in the past year?’. Mental stress was classified into the four categories of severe, high, moderate, or low.

Laboratory parameters were obtained at the health check site. Hypertension was defined as a systolic blood pressure ≥140 mm Hg or diastolic blood pressure ≥90 mm Hg, or the use of antihypertensive medications. Subjects with a body mass index ≥25.0 kg/m^2^ were categorized as obese. Presence of diabetes was defined as a plasma glucose level ≥126 mg/dL, hemoglobin A1c ≥6.5% (Japanese Diabetes Society value), or the use of antidiabetic medications.

The death code (International Classification of Disease, 10th Revision) and the date and place of death were reviewed from death certificates. Incidences of cardiovascular disease were reviewed from the Yamagata Stroke and Acute Myocardial Infarction (AMI) registries and the Yamagata Society in Treatment for Cerebral Stroke (YSTCS).

### Statistical analysis

Data are expressed as the mean (standard deviation) for continuous values and as percentages of the total number of subjects for categorical variables. Analysis of variance was performed to evaluate differences in mean values and the chi-squared test was utilized to evaluate differences in proportions. Kaplan-Meier analysis with log-rank test and both unadjusted and adjusted Cox-proportional hazard model analyses were performed to examine relationships between frequency of laughter and both all-cause mortality and cardiovascular disease incidence. In the multivariate-adjusted model, the hazard ratio (HR) was adjusted for age, gender, hypertension, diabetes, obesity, alcohol drinking status, and smoking status. Some cases were excluded from multivariate analyses due to a lack of clinical parameters. To examine whether the association between laughter and prognosis differed by background characteristics, we performed subgroup analyses. *P* values <0.05 were defined as statistically significant. All statistical analyses were performed using JMP version 14 software (SAS Institute, Cary, NC, USA).

## RESULTS

Participants comprised 7,003 males (40.8%) and 10,149 females (59.2%), with a mean age of 62.8 years. Prevalence of subjects with a frequency of laughter ≥1/week, ≥1/month but <1/week, and <1/month were 14,096 (82.2%), 2,486 (14.5%), and 570 (3.3%), respectively. Baseline characteristics of study subjects according to the frequency of laughter are shown in Table 1. In subjects with a low frequency of laughter, physical inactivity and proportions of males, present smokers, those with diabetes, single, and physical inactivity were significantly higher than in subjects showing a high frequency of laughter.

**Table 1.** Characteristics of study subjects

	Laughter ≥1/week	≤1/month but ≥1/week	<1/month	*P* value
Number of subjects, %	14,096 (82.2)	2,486 (14.5)	570 (3.3)	
Age, years, mean (SD)	62.6 (8.4)	63.7 (7.6)	62.6 (8.1)	<0.001
Male gender, %	5,231 (37.1)	1,406 (56.6)	365 (64.0)	<0.001
BMI, kg/m^2^, mean (SD)	23.1 (3.2)	23.1 (3.2)	23.0 (3.3)	0.884
Smoking, %				
Current	1,158 (11.6)	392 (16.6)	104 (18.8)	
Past	3,377 (25.2)	763 (32.3)	202 (36.5)	
Never	8,480 (63.2)	1,206 (51.1)	247 (44.7)	<0.001
Drinker, %				
Current	7,461 (54.3)	1,462 (60.4)	327 (59.0)	
Past	301 (2.2)	79 (3.3)	24 (4.3)	
Never	5,974 (43.5)	879 (36.3)	203 (36.6)	<0.001
Medical history, %				
Hypertension	3,608 (25.6)	686 (27.6)	142 (25.9)	0.096
Diabetes	1,186 (8.41)	254 (10.2)	60 (10.5)	0.004
Physical activity, %				
Exercise				
≥1 time/week	9,576 (71.6)	1,553 (66.3)	315 (59.4)	
Rarely	1,807 (13.5)	387 (16.5)	78 (14.7)	
None	1,993 (14.9)	402 (17.2)	137 (25.6)	<0.001
Education, %				
Primary or junior high school	2,048 (14.9)	430 (17.7)	88 (15.9)	
High school	7,511 (54.8)	1,393 (57.4)	295 (53.2)	
College or higher education	4,115 (30.3)	606 (25.0)	172 (31.0)	<0.001
Marital status, %				
Living with a spouse	11,545 (93.7)	1,917 (90.5)	377 (83.6)	
Single, divorced, or widowed	772 (6.3)	202 (9.5)	74 (16.4)	<0.001
Perceived mental stress, %				
Low	565 (4.2)	109 (4.6)	43 (7.8)	
Moderate	3,457 (25.5)	537 (22.6)	115 (20.9)	
High/Severe	9,521 (70.3)	1,733 (72.9)	393 (71.3)	<0.001
Frequency of social participation, %				
≥1/week	803 (6.0)	134 (5.7)	28 (5.2)	
≥1/month but <1/week	2,844 (21.2)	491 (20.8)	105 (19.4)	
<1/month	9,768 (72.8)	1,740 (73.6)	409 (75.5)	<0.001

BMI, body mass index; SD, standard deviation.

**Figure 1.**
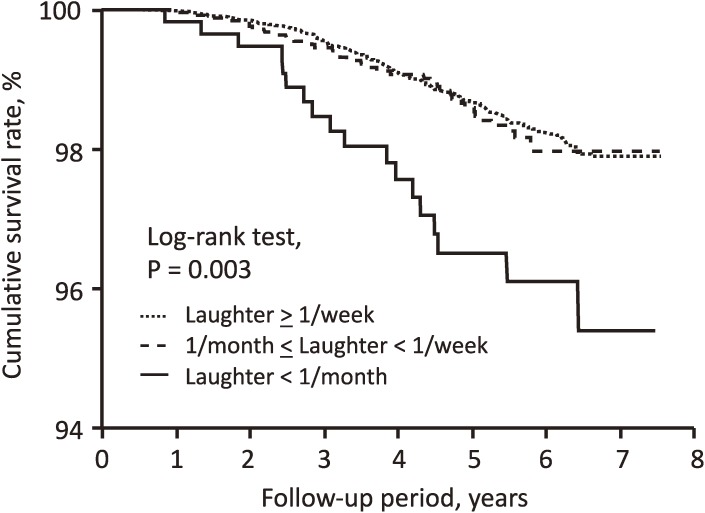
Kaplan-Meier analysis of overall survival according to the frequency of laughter

**Figure 2.**
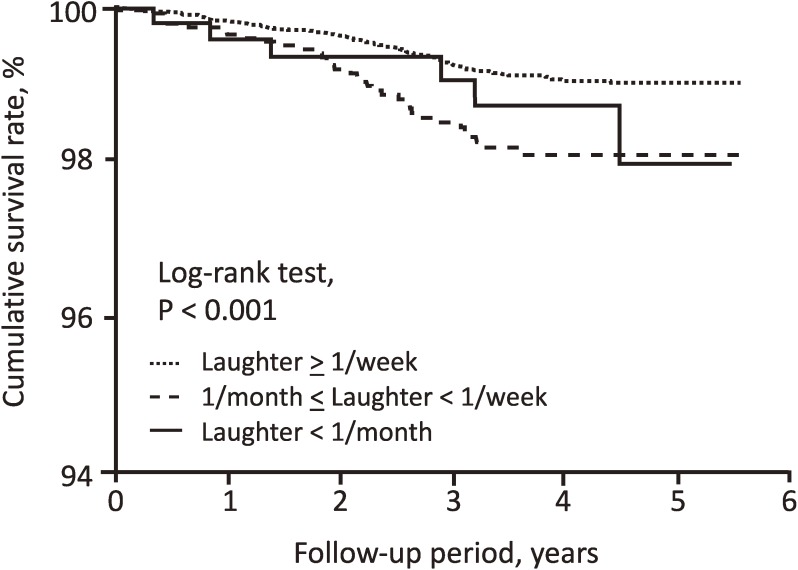
Kaplan-Meier analysis of cardiovascular disease-free survival according to the frequency of laughter

During follow-up (median 5.4 years), a total of 257 (1.5%) deaths and 138 (0.8%) cardiovascular disease incidences were recorded. We compared mortality and cardiovascular disease incidences by daily frequency of laughter using the Kaplan-Meier method. All-cause mortality was significantly higher in subjects with a low frequency of laughter (log-rank test, *P* = 0.003) (Figure 1). A similar curve was observed for cardiovascular disease incidence (log-rank test, *P* < 0.001) (Figure 2).

Next, we examined independent associations of the daily frequency of laughter with all-cause death and cardiovascular disease incidence using Cox-proportional hazard analyses (Table 2). In an unadjusted model, all-cause mortality was significantly higher among individuals who laughed <1/month (HR 2.38; 95% CI, 1.42–3.74) than in those who laughed ≥1/week. After adjusting for age, gender, hypertension, diabetes, smoking status, and alcohol drinking status, risk of all-cause mortality was significantly higher in subjects who laughed <1/month than in subjects who laughed ≥1/week (HR 1.95; 95% CI, 1.16–3.09). Risk of cardiovascular disease incidence was significantly higher in subjects who laughed ≥1/month but <1/week than in subjects who laughed ≥1/week (HR 2.06; 95% CI, 1.38–3.00) in the unadjusted model. After adjusting for the above-mentioned potential confounders, cardiovascular disease incidence was significantly higher in subjects who laughed ≥1/month and <1/week than in subjects who laughed ≥1/week (HR 1.62; 95% CI, 1.07–2.40).

**Table 2.** Cox proportional analysis: associations between frequency of laughter and mortality and cardiovascular events

(Number of cases/Number of samples)	Unadjusted			Age-, gender-adjusted			Multivariate-adjusted^a^		

	HR	95% CI	*P* value	HR	95% CI	*P* value	HR	95% CI	*P* value
All-cause mortality									
≥1/week (*n* = 202/14,096)	reference			reference			reference		
≥1/month but <1/week (*n* = 37/2,486)	0.81	0.73–1.64	0.805	0.85	0.59–1.19	0.344	0.81	0.55–1.15	0.249
<1/month (*n* = 18/570)	2.38	1.42–3.74	0.002	1.93	1.15–3.06	0.015	1.95	1.16–3.09	0.014

Cardiovascular event and mortality									
≥1/week (*n* = 97/14,096)	reference			reference			reference		
≥1/month but <1/week (*n* = 35/2,486)	2.06	1.38–3.00	<0.001	1.63	1.08–2.39	0.018	1.62	1.07–2.40	0.023
<1/month (*n* = 6/570)	1.66	0.64–3.48	0.261	1.35	0.52–2.85	0.49	1.42	0.55–3.00	0.427

CI, confidence interval; HR, hazard ratio.

^a^Adjusted for age, gender, hypertension, diabetes, smoking and alcohol consumption.

We then performed subgroup analyses for associations between frequency of laughter and all-cause mortality (Table 3). After adjustment for age, hypertension, diabetes, alcohol drinking status, smoking status, significantly higher HRs for all-cause mortality were seen in subjects who laughed <1/month, compared to subjects who laughed ≥1/week, among subgroups, such as female, non-hypertensives, nondiabetics, obese, moderate level of perceived mental stress, and graduation for college or higher. As shown in Table 1, the prevalence of low mental stress was higher in the least laughter group (7.8%) compared with the other groups (4.2–4.7%), while the prevalence of high/severe mental stress in the least laughter group was similar to those of other groups (70.3–72.9%). This indicates that a low frequency of laughter did not always reflect a mental stress-free status in this study.

**Table 3.** Subgroup analysis: association between frequency of laughter and all-cause of mortality

(Number of cases/Number of samples)	≥1/week	≥1/month but <1/week			<1/month		

	HR	HR	95% CI	*P* value	HR	95% CI	*P* value
Men (*n* = 163/7,003)	1.0	0.78	0.50–1.17	0.244	1.53	0.80–2.66	0.184
Women (*n* = 94/10,149)	1.0	0.88	0.42–1.82	0.723	3.98	1.72–9.21	0.001
<65 years (*n* = 81/8,713)	1.0	1.10	0.61–1.97	0.757	0.61	0.15–2.52	0.500
≥65 years (*n* = 176/8,439)	1.0	0.69	0.43–1.11	0.123	2.75	1.63–4.66	0.002
Hypertension							
No (*n* = 172/12,716)	1.0	0.61	0.37–1.01	0.056	2.31	1.34–3.97	0.002
Yes (*n* = 85/4,436)	1.0	1.22	0.69–2.05	0.478	1.04	0.25–2.84	0.942
Diabetes							
No (*n* = 216/15,652)	1.0	0.71	0.47–1.09	0.119	2.07	1.23–3.48	0.006
Yes (*n* = 41/1,500)	1.0	1.32	0.59–2.70	0.475	1.28	0.20–4.28	0.742
Obesity							
No (*n* = 190/11,531)	1.0	0.68	0.43–1.06	0.092	1.65	0.91–2.99	0.099
Yes (*n* = 61/3,875)	1.0	1.19	0.58–2.25	0.623	3.20	1.21–7.10	0.022
Alcohol intake							
Never (*n* = 81/7,056)	1.0	0.64	0.26–1.30	0.229	2.46	1.00–5.10	0.048
Past (*n* = 13/404)	1.0	0.51	0.03–2.94	0.503	10.70	2.43–45.9	0.003
Current (*n* = 157/9,250)	1.0	0.90	0.57–1.35	0.612	1.16	0.49–2.31	0.713
Smoking							
Never (*n* = 105/9,933)	1.0	0.68	0.32–1.25	0.22	2.16	0.83–4.59	0.106
Past (*n* = 91/4,342)	1.0	0.83	0.44–1.42	0.506	2.52	1.22–4.69	0.015
Current (*n* = 51/2,054)	1.0	0.98	0.46–1.91	0.964	0.80	0.13–2.62	0.752
Education							
Primary or junior high school (*n* = 42/2,566)	1.0	0.62	0.21–1.47	0.301	1.85	0.44–5.26	0.350
High school (*n* = 131/9,199)	1.0	0.82	0.50–1.35	0.437	1.60	0.78–3.32	0.203
College or higher education (*n* = 74/4,923)	1.0	1.01	0.48–1.92	0.197	2.69	1.10–5.64	0.032
Marital status							
Living with a spouse (*n* = 197/13,839)	1.0	0.63	0.40–1.00	0.048	1.21	0.59–2.48	0.594
Single, divorced, or widowed (*n* = 23/1,048)	1.0	1.25	0.39–3.45	0.683	2.53	0.69–7.46	0.148
Perceived mental stress							
Low (*n* = 18/717)	1.0	0.98	0.28–3.44	0.975	0.88	0.11–6.81	0.904
Moderate (*n* = 83/4,109)	1.0	0.76	0.35–1.46	0.453	3.23	1.53–8.65	0.002
High/Severe (*n* = 147/11,647)	1.0	0.84	0.52–1.35	0.467	1.53	0.74–3.15	0.253

CI, confidence interval; HR, hazard ratio.

Adjusted for age, gender, hypertension, diabetes, smoking and alcohol consumption.

We did not perform subgroup analyses for associations between frequency of laughter and cardiovascular disease, because only six cases of cardiovascular disease occurred in subjects who laughed <1/month during the follow-up period.

## DISCUSSION

This prospective study examined associations between frequency of laughter and all-cause mortality and cardiovascular events. Results showed that low frequency of laughter was independently associated with all-cause mortality and cardiovascular disease incidence in a Japanese general population.

In our study, the baseline characteristics showed that low frequency of laughter was associated with higher prevalences of male, current drinker, diabetes, low physical activity, and living without a spouse. These findings are consistent with the results of previous reports,^8^^,^^15^ indicating that subjects with a low frequency of laughter are likely to possess multiple risk factors for cardiovascular disease.

Of note, our study revealed that the frequency of laughter was independently associated with all-cause mortality and cardiovascular disease incidence, even after adjustments for age, gender, and multiple well-known risk factors, such as smoking, alcohol drinking status, hypertension, and diabetes. Previous cross-sectional reports disclosed that the daily frequency of laughter was associated with the prevalence of cardiovascular diseases, after adjusting for established risk factors.^8^ Based on previous findings and our present results, we suggest that laughter itself contributes to increasing longevity and reducing cardiovascular disease incidence, independent of established risk factors.

During follow-up, 257 subjects died and 138 subjects experienced cardiovascular events among all subjects. The number of all-cause deaths was 202 with most laughter, 37 with middle laughter, and 18 with least laughter, while the number of cardiovascular events was 97 with most laughter, 35 with middle laughter, and 6 with least laughter. We assume that one reason for the different results between all-cause mortality and cardiovascular events in our study is that the number of cardiovascular events in the least laughter group was relatively low.

The mechanisms underlying the preventive effects of the frequency of laughter on all-cause mortality and cardiovascular disease remain unclear, although several possible explanations may be suggested. First, laughter may be associated with health-promoting behaviors. In this study, the group of subjects with a higher frequency of laughter showed lower proportions of current smokers and current drinkers, and low physical activity. Furthermore, with regard to the immune system, laughter has been shown to affect a range of immunological factors. Previous studies have shown that laughter increases levels of immunoglobulins A, G, M, and complement C3, and natural killer cell activity.^16^^,^^17^ In addition, laughter improves vascular endothelial function and arterial stiffness^18^^,^^19^ and lowers the increase in postprandial blood glucose^20^ and salivary chromogranin A, a biomarker for stress.^21^ Such findings suggest that laughter might affect prognosis by modulating various physical functions.

Interestingly, subgroup analysis disclosed that the effect of daily frequency of laughter was significant among females, but not among males. Generally, males tend not to exhibit their emotions, and in this study, the frequency of laughing out loud was lower in males than in females. This may have attenuated the association between frequency of laughter and prognosis in males. In addition, the effect of daily frequent laughter was stronger among the elderly, those with obesity and those with moderate stress levels. A higher HR for all-cause mortality was observed at the moderate stress level compared with other stress levels. As shown Table 1, the prevalence of low mental stress was higher in the least laughter group (7.8%) compared with other groups (4.2–4.7%), and the prevalence of high/severe mental stress in the least laughter group was similar with those of other groups (70.3–72.9%). This indicates that a low frequency of laughter did not always reflect a mental stress-free status in this study. Previous studies have reported that a higher HR for cancer incidence was observed at increased stress levels.^22^ No clear explanation for this finding has yet been provided, suggesting that the influence of laughter might be more pronounced in some subpopulations with elevated risk for cardiovascular disease and malignancies.

The strengths of the present study were its prospective design and large sample size. Furthermore, adjustment was performed using multiple well-known risk factors such as age, gender, hypertension, diabetes, smoking status, and alcohol drinking status. Conversely, several limitations of this study must also be considered. First, the daily frequency of laughter was evaluated from a simple single question: “How often do you laugh out loud?”. We did not take into account different types of laughter or degree. In this study we defined “laugh out loud” as laughter; accordingly, silent laughing and smiling were not counted as laughter. In this setting, the frequency of laughter might be underestimated. However, given that the degree of laughter varies among people, we assume that this might have caused both the overestimation and underestimation of laughter. Second, we assessed the frequency of laughter at a single baseline survey, and did not evaluate changes in the frequency of laughter. However, Hirosaki et al reported that the 1-year test-retest reliability of this variable in a previous study using the Spearman correlation coefficient was 0.61 (*P* < 0.001).^23^ Third, we performed multivariate analyses adjusting for various potential confounding factors. Nevertheless, unknown confounding factors may that were not accounted for may still have been present. Further, because of the small number of events, particularly in the least laughter group, we did not include analyses with additional adjustment in this study. Fourth, theoretically, the AMI registries and YSTCS covered almost all incidences of cardiovascular disease in the area. However, it is possible that some cases of asymptomatic cardiovascular event were not recorded. Fifth, the study subjects were participants in a community-based annual health checkup. Compared to the general population, they might have been more health-conscious and had a higher level of social activity. Therefore, a degree of selection bias might also be present.

Allowing for these limitations, our findings support the positive effects of laughter therapy, such as laughter yoga for longevity. Laughter therapy is easily accessible and acceptable, and is not cost-prohibitive. These findings support the wide use of laughter therapy in the general population.

### Conclusion

This study identified the daily frequency of laughter as an independent risk factor for all-cause mortality and cardiovascular disease incidence among a general Japanese population. Our findings suggest that increasing the frequency of laughter might reduce the risk of cardiovascular disease and increase longevity.
